# Kinematic Patterns of Different Loading Profiles Before and After Total Knee Arthroplasty: A Cadaveric Study

**DOI:** 10.3390/bioengineering11111064

**Published:** 2024-10-24

**Authors:** Saskia A. Brendle, Sven Krueger, Janno Fehrenbacher, Joachim Grifka, Peter E. Müller, William M. Mihalko, Berna Richter, Thomas M. Grupp

**Affiliations:** 1 Research & Development, Aesculap AG, 78532 Tuttlingen, Germany; 2 Department of Orthopaedic and Trauma Surgery, Musculoskeletal University Center Munich (MUM), Campus Grosshadern, LMU Munich, 81377 Munich, Germany; 3 Department of Mechanical and Process Engineering, Offenburg University of Applied Sciences, 77652 Offenburg, Germany; 4 Department of Orthopaedics, Asklepios Klinikum, 93077 Bad Abbach, Germany; 5 Campbell Clinic, Department of Orthopaedic Surgery & Biomedical Engineering, University of Tennessee Health Science Center, Memphis, TN 38104, USA

**Keywords:** knee, biomechanics, cadaveric study, kinematics, TKA design

## Abstract

One of the major goals of total knee arthroplasty (TKA) is to restore the physiological function of the knee. In order to select the appropriate TKA design for a specific patient, it would be helpful to understand whether there is an association between passive knee kinematics intraoperatively and during complex activities, such as ascending stairs. Therefore, the primary objective of this study was to compare the anterior–posterior (AP) range of motion during simulated passive flexion and stair ascent at different conditions in the same knees using a six-degrees-of-freedom joint motion simulator, and secondary, to identify whether differences between TKA designs with and without a post-cam mechanism can be detected during both activities, and if one design is superior in recreating the AP translation of the native knee. It was shown that neither TKA design was superior in restoring the mean native AP translation, but that both CR/CS and PS TKA designs may be suitable to restore the individual native kinematic pattern. Moreover, it was shown that passive and complex loading scenarios do not result in exactly the same kinematic pattern, but lead to the same choice of implant design to restore the general kinematic behavior of the native individual knee.

## 1. Introduction

Total knee arthroplasty (TKA) is one of the most common procedures for the treatment of severe knee osteoarthritis [1,2,3]. Even though knee prostheses have improved greatly and became one of the most reliable joint replacements, numerous studies point out that only approximately 80% of the patients are satisfied with the results of their TKA [4,5,6]. It is hypothesized that the ability of the prosthesis to recreate the native tibiofemoral kinematics, especially the femoral rollback, is beneficial regarding patient satisfaction after TKA [7,8]. However, previous studies showed a high variability in the native knee kinematics, indicating the necessity for various TKA designs in order to restore physiological knee function [9]. For posterior cruciate ligament (PCL)-deficient TKA, surgeons can choose between inlay designs with or without a post-cam mechanism. It was shown that both inlay designs provide similar stability, but more potential complications were reported with the post-cam mechanism [10,11,12]. In addition, the post-cam mechanism could have an effect on tibiofemoral kinematics and result in a more pronounced femoral rollback [11,12].

Modern surgical navigation and robotic systems allow intraoperative measurement of knee joint kinematics and stability before and after implantation and provide real-time feedback on multiple parameters to adjust implant type, positioning and overall alignment during surgery [8,13,14,15,16,17,18,19]. Therefore, the intraoperative kinematic analysis may assist in the selection of the TKA design and help surgeons get closer in restoring physiological knee function, since the only opportunity for adjustment of the kinematics is during surgery. However, it is not clear whether the differences in anterior–posterior (AP) translation between the native condition and different TKA designs can be detected intraoperatively during passive movements [13,17]. Furthermore, it is necessary to understand whether there is an association between passive knee kinematics measured during surgery and knee kinematics during complex activities of daily living. A previous study of Grassi et al. compared flexion–extension actively performed by the patient and passively performed by the surgeon using a navigation system [18]. They found that muscle contraction did not significantly affect the knee kinematics before and after TKA. Kono et al. reached the same conclusion in a cadaveric study [13]. Belvedere et al. showed that passive knee kinematics measured intraoperatively after TKA using a navigation system were predictive of postoperative kinematics measured using monoplane fluoroscopy during several complex weight-bearing activities [15,16]. Gasparutto et al. compared the passive knee kinematics during surgery with active knee kinematics during walking [14]. However, they could not draw any solid conclusions about differences between the activities because of too many influencing factors, such as the use of different methods to measure the kinematics during different activities. This shows the importance of a highly controlled environment for valid comparison between different conditions and loading profiles.

Therefore, the primary objective of this study was to compare the AP range of motion and position of the medial and lateral femoral condyles during passive flexion and a complex activity of daily living such as stair ascent, in the native condition, after resection of the cruciate ligaments and after implantation of two different TKA designs (with and without a post-cam mechanism) in the same knees. The secondary objective was to identify whether differences between the two different TKA designs can be detected during passive flexion and stair ascent, and if one design is superior in recreating the AP translation of the native knee. The hypothesis was that passive and complex loading scenarios show a comparable range of motion in the AP direction, especially after TKA. We anticipated that with the post-cam TKA design, the femoral posterior translation is higher compared to the TKA design without a post-cam mechanism. Furthermore, we hypothesized that no design is superior in restoring the mean native AP translation but for individual knees either the TKA design with or without a post-cam mechanism is superior in recreating the individual native kinematic pattern.

## 2. Materials and Methods

### 2.1. Specimen Preparation

This in vitro study used thirteen fresh-frozen human cadaveric lower right extremities, preserved from the femoral head to the malleoli. The samples included three females and ten males with a mean age of 67 ± 10 years and a mean body mass index of 23.3 ± 8.0 kg/m^2^. Medical records did not indicate any pre-existing knee disorders, surgical interventions, or other relevant pathologies. No specimen had a severe varus or valgus deformity (≥10°). The mean native anatomical slope medially was 9.1 ± 2.8°. Ethical approval was obtained from the ethics committee of the Ludwig Maximilian University of Munich (No. 20-0856).

The specimen preparation and the general experimental setup were based on a methodology recently published by Brendle et al. [20]. Before the experiment, landmark-based femoral and tibial coordinate systems were generated by using segmented computed tomography (CT) scans of the specimens. Each specimen was thawed for 24 h at 7 °C and then prepared for testing. The proximal and distal segments of the leg were skeletonized, preserving soft tissue 100 mm superior and 50 mm inferior to the knee joint space. GOM measuring points (1.5 mm, Carl Zeiss GOM Metrology GmbH, Braunschweig, Germany) were attached to the clean bones and 3D fittings of femur and tibia were performed by aligning the segmented CT scans to the previously acquired 3D point clouds of each bone (ARAMIS 12M, Carl Zeiss GOM Metrology GmbH, Braunschweig, Germany). Subsequently, the 3D fitting information was saved and the femur and tibia were transected and embedded in custom-made aluminum pots using fast-cast resin (Gößl und Pfaff, Brautlach, Germany) to allow fixation in a six-degrees-of-freedom joint motion simulator. Thereby, the femur was embedded such that the femoral coordinate system was aligned with the upper coordinate system of the joint motion simulator, whereas the tibia was embedded to achieve 0° flexion.

### 2.2. Implantation

**Figure 1:**
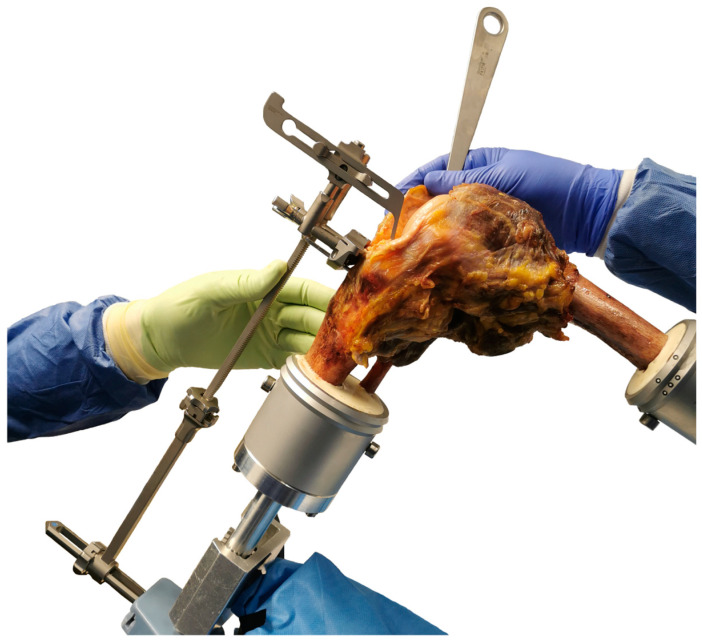
Extramedullary alignment technique for the tibial cut with reconstruction of the center of the malleoli.

The specimens underwent cruciate-sacrificing TKA without patella resurfacing by an experienced knee surgeon using oneKNEE TKA components (Aesculap AG, Tuttlingen, Germany). This TKA system allows the use of a femoral component and an inlay with or without a post-cam mechanism (PS or CR/CS) fixed to the same tibial component. First, a standard medial parapatellar arthrotomy and lateral dislocation of the patella were performed. After resection of osteophytes and menisci, a tibial cut was made using an extramedullary alignment guide. This was possible since the exact positions of the tibia and fibula in the tibia pot were known from the previous 3D fitting, and therefore the center of the malleoli could be reconstructed using custom-made fixtures specifically designed to match the alignment guide as shown in Figure 1.

The tibial cut was made with a 0° slope, whereas a 3° posterior slope is integrated in the polyethylene inserts. The medial third of the tuberosity was used as a reference for the tibial rotation alignment. The femoral cut was made perpendicular to the mechanical axis of the femur using an intramedullary guide. Rotation of the femoral component was aligned as indicated by the soft-tissue strain at 90° knee flexion. Furthermore, a box preparation was conducted to allow the use of a PS femoral component. The trial components were positioned and the ligamentous situation was evaluated in flexion and extension to ensure proper implant alignment, knee balance, and function. Following this, the definite CR/CS components underwent a 3D fitting by aligning their computer-aided design (CAD) files to 3D point clouds of the femoral and tibial component. Afterwards, the components were inserted and the positions of the implants relative to the respective bones were measured (ARAMIS 12M, Carl Zeiss GOM Metrology GmbH, Braunschweig, Germany). In a second step, the CR/CS femoral component and inlay were changed by a PS femoral component and inlay of the same size. The bone cuts remained unchanged.

### 2.3. Experimental Testing

**Figure 2:**
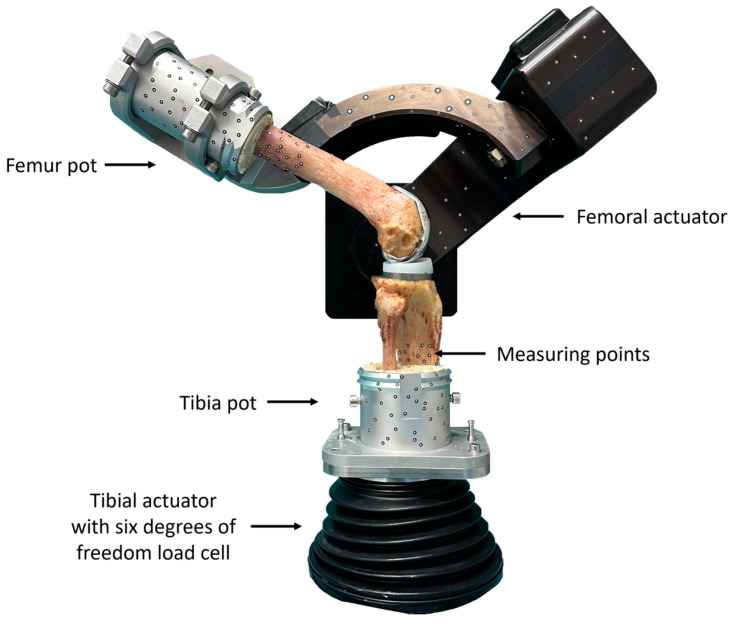
Experimental setup with the instrumented knee specimen mounted on the six-degrees-of-freedom joint motion simulator at 60° flexion. Soft tissue was removed for clarity, but left intact during actual testing.

Testing was performed on a six-degrees-of-freedom joint motion simulator (VIVO, Advanced Mechanical, Technologies Inc., Watertown, MA, USA), as illustrated in Figure 2. The femoral actuator provides flexion–extension and varus–valgus rotations, whereas the tibial actuator manages medial–lateral, anterior–posterior and proximal–distal translations, and internal-external rotations. The simulator allows independent control of each degree-of-freedom in either force or displacement mode. The forces and motions are expressed following the Grood and Suntay conventions [21].

**Figure 3:**
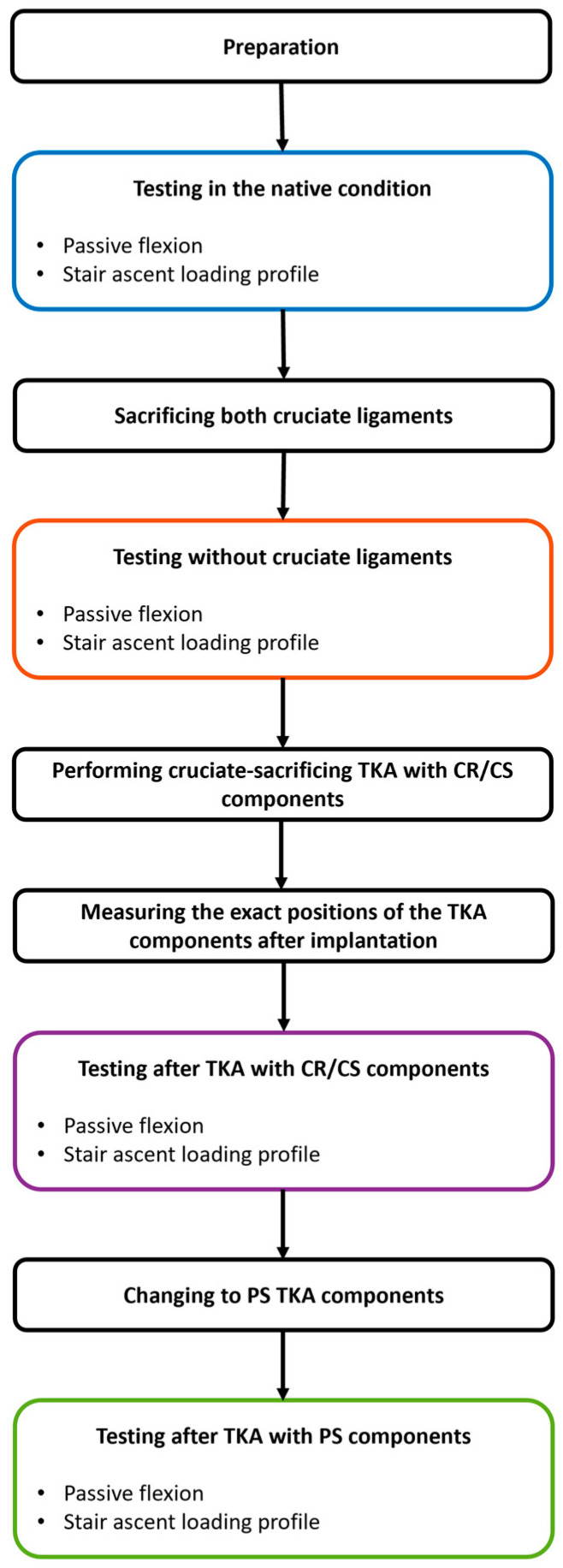
Illustration of the key steps of the entire testing process. TKA = total knee arthroplasty. CR/CS = TKA design without a post-cam mechanism. PS = TKA design with a post-cam mechanism.

After mounting the native specimen in the joint motion simulator, the previously generated 3D fitting information of the segmented CT scans were again projected onto the residual bones using the measuring points. In this way, the 3D information of the complete femur and tibia were available even after parts of the bones were resected. Based on this, the relative position of the femoral and tibial coordinate systems in the native condition was recorded and transferred to the joint motion simulator. Afterwards, the specimens were subjected to dynamic testing. The neutral path of motion of each knee was recorded by applying continuous knee flexion from 0° to 90° while maintaining an axial compression force of 50 N and all other forces/moments at 0 N/Nm. In addition, stair ascent was simulated by applying AVER75 loading data obtained from the Orthoload database [22]. The loads were reduced by 75% to prevent specimen damage considering the absence of muscle activity. The flexion angle was prescribed and all other degrees-of-freedom were controlled in force mode. Each loading profile was applied in the native condition, after resection of the cruciate ligaments and with both a CR/CS and a PS TKA design for four cycles at a frequency of 0.04 Hz. For all conditions, the knee capsule was opened using a medial parapatellar approach and closed with surgical sutures (Number 1 Vicryl, B. Braun, Melsungen, Germany). During testing, the relative position of the femoral and tibial coordinate systems was recorded by the joint motion simulator. To mitigate the effects of tissue drying during testing, the specimens were kept moist with sodium chloride solution. Furthermore, the passive tension of the patella tendon was simulated by a spring sutured to the quadriceps tendon with an increasing force of up to 50 N at 90° flexion. Figure 3 illustrates the entire testing process.

### 2.4. Data Analysis

Data from a single cycle of each experiment were analyzed using MATLAB (Version R2023a, MathWorks Inc., Natick, MA, USA) [20]. Statistical analyses were performed using Minitab (Version 21.2, Minitab GmbH, Munich, Germany). For all analyses, the medial and lateral flexion facet centers (MFC and LFC) of the native femoral condyles were projected onto the tibial plane at different timepoints. Subsequently, the positions were normalized to the anterior–posterior and medial–lateral width of the articular surface of the respective tibia, which were defined as the distance between the most anterior and posterior points of the medial articular surface and the most medial and lateral points of the proximal tibia, respectively. Thus, irrespectively of the different tibia size: 0 and 1 correspond to the most posterior and most anterior position, respectively.

The normalized AP range of motion (ROM) medially and laterally was analyzed for both movements in all conditions between 14° and 90° of flexion, as the flexion angles of the two loading profiles overlap in this range and, therefore, only the influence of the load situation and not that of the flexion angle was investigated. The significance of the differences between the normalized medial and lateral AP ROM during passive knee flexion and stair ascent was investigated for all conditions using Wilcoxon signed-rank tests with the level of significance set at *p* ≤ 0.05. In addition, the projections of the MFC and LFC of all specimens at 14° flexion and at flexion angles between 15° and 90° are plotted in 5° increments on a normalized tibia for all loading profiles and conditions.

To investigate the differences between the native condition and the two TKA designs, the normalized medial and lateral AP translation of each specimen was calculated for the entire loading profiles in the different conditions and normalized to the native positions at 0° during passive flexion. Wilcoxon signed-rank tests were used to compare the normalized AP translation between the native condition, and after implantation of a CR/CS and a PS TKA design pairwise at 5° flexion intervals for passive flexion and 5 % gait cycle intervals for stair ascent (*p* ≤ 0.05). Furthermore, the projections of the MFC and LFC of three exemplary specimens are shown for the full range of flexion of both loading profiles in the native condition, and with both TKA designs at 5° flexion and 5% gait cycle intervals, respectively.

Four specimens showed joint luxation or exceeded the travel limits of the joint motion simulator during one of the applied loading scenarios and were therefore removed from the data analysis.

## 3. Results

### 3.1. Anterior–Posterior Range of Motion and Position During Passive Flexion and Stair Ascent

**Figure 4:**
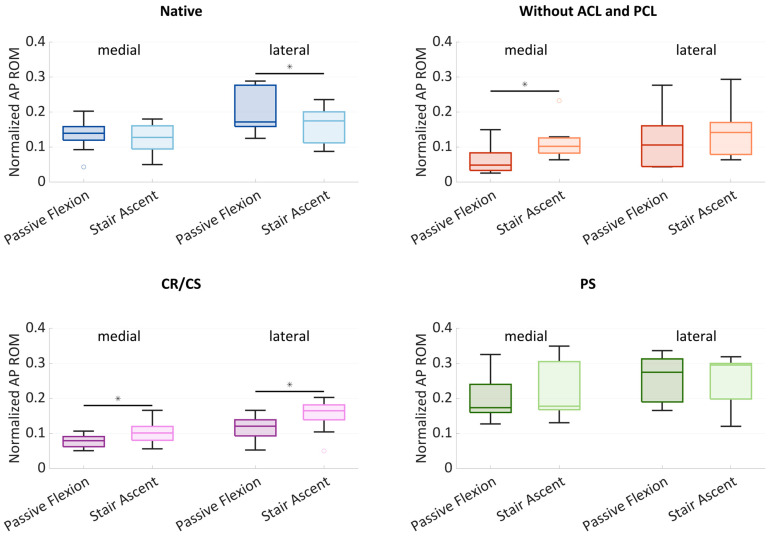
Normalized medial and lateral anterior–posterior (AP) range of motion (ROM) during passive flexion and stair ascent (*n* = 9) in the native condition (blue), after resection of the cruciate ligaments (red) and after implantation of the CR/CS (purple) and PS (green) total knee arthroplasty (TKA) design. Significant differences are marked with an asterisk (*p* ≤ 0.05).

Figure 4 shows boxplots of the normalized AP ROM medially and laterally. The boxplots include the median normalized AP ROM, the first and third quartiles, and the range. Outliers are shown with dots. Significant differences (*p* ≤ 0.05) are marked with an asterisk.

In the native condition, the median medial AP ROM was 0.14 during passive flexion and slightly lower with 0.13 during stair ascent. The median lateral AP ROM showed a higher variability and was greater than the medial ROM for both loading profiles. Furthermore, there was a significant difference (*p* = 0.024) between the AP ROM during passive flexion (0.17) and during stair ascent (0.18). After cruciate ligament resection, the median AP ROM was generally smaller compared to the native condition. The median medial AP ROM was significantly higher (*p* = 0.013) at 0.10 during stair ascent, compared to 0.05 during passive flexion. The median lateral AP ROM was higher than the median medial AP ROM with 0.11 during passive flexion and 0.14 during stair ascent. After implantation of the CR/CS TKA design, both the medial and lateral median AP ROM were significantly different between passive flexion and stair ascent (*p* = 0.013 and *p* = 0.033). Compared to the condition without cruciate ligaments, the median medial AP ROM increased slightly during passive flexion (0.08) and remained the same during stair ascent (0.10). The median lateral AP ROM increased during both passive flexion (0.12) and stair ascent (0.17) and was greater than the medial AP ROM for both loading profiles. After implantation of the PS TKA design, the medial and lateral AP ROM were higher compared to all other conditions. The median medial AP ROM was 0.17 during passive flexion and 0.18 during stair ascent, whereas the median lateral AP ROM was 0.28 and 0.30, respectively.

In all conditions, a generally higher AP ROM was found laterally, which can be interpreted as a medial pivoting kinematic. In addition, the lateral variability was greater in the native condition and without cruciate ligaments, which may be due to a more or less pronounced medial pivoting characteristic.

**Figure 5:**
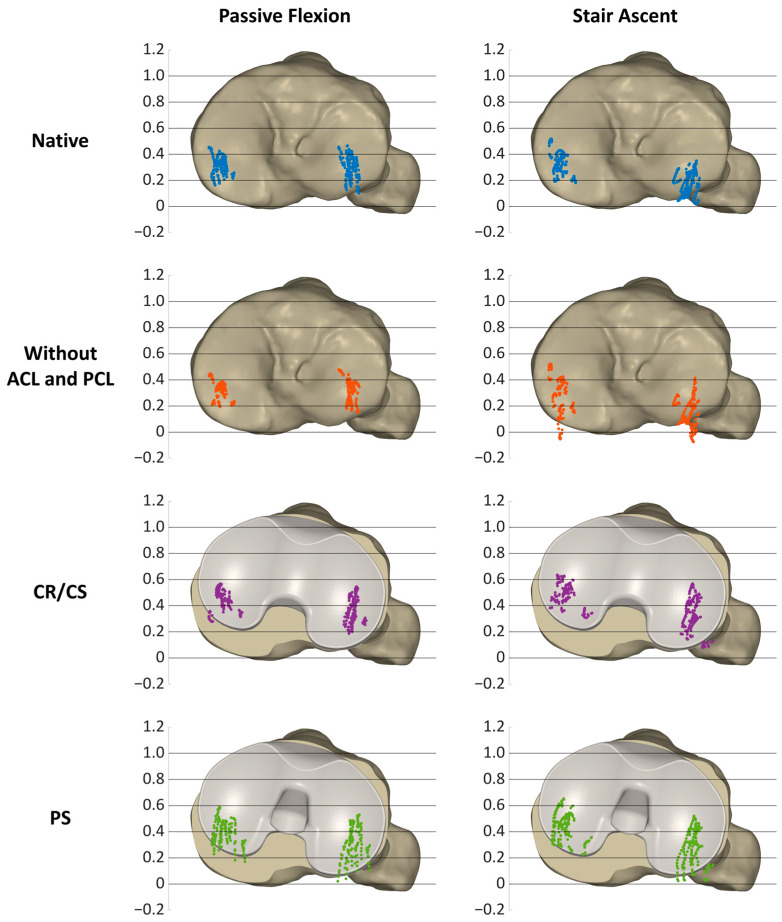
Projections of the native medial and lateral flexion facet centers (MFC and LFC) of all specimens (*n* = 9) and all timepoints during passive flexion and stair ascent on a normalized tibia.

Figure 5 shows the projections of the MFC and LFC of all specimens at 14° of flexion and at flexion angles between 15° and 90° in 5° increments on a normalized tibia for all loading profiles and conditions, illustrating the condylar motion patterns of the specimens.

In the native condition, the medial condyles ranged around the posterior third of the tibia, mainly between 0.5 and 0.15 for both passive flexion and stair ascent. The lateral condyles not only showed a difference in AP ROM between passive flexion and stair ascent, but also a difference in position. During passive flexion, the lateral condyles were mainly between 0.5 and 0.1, but slightly more posterior between 0.4 and 0 during stair ascent. After resection of the cruciate ligaments, the projections of both condyles resulted in a high density of points during passive flexion, ranging mainly between 0.4 and 0.2 medially and between 0.5 and 0.15 laterally. In contrast, during stair ascent, the projections of the MFC and LFC showed a high variability and were mainly located between 0.5 and −0.05 medially, and slightly more posterior, between 0.4 and −0.05 laterally. After implantation of the CR/CS TKA design, the projections of the medial condyles were located more anterior than in the native condition and after cruciate ligament resection during both activities, ranging mainly between 0.6 and 0.3 during passive flexion and between 0.65 and 0.3 during stair ascent. The projections of the lateral condyles were also located more anterior than in the native condition, especially during stair ascent (0.6–0.1). After implantation of the PS TKA design, the anterior border of the point pattern was approximately in the same position as for the CR/CS TKA design for both condyles and loading profiles. In contrast, the posterior border of the point pattern was located more posteriorly compared to the CR/CS design. The pattern during passive flexion and stair ascent was comparable, ranging mainly between 0.6 and 0.2 medially and between 0.5 and 0 laterally.

### 3.2. AP Translation in the Native Condition and After TKA

**Figure 6:**
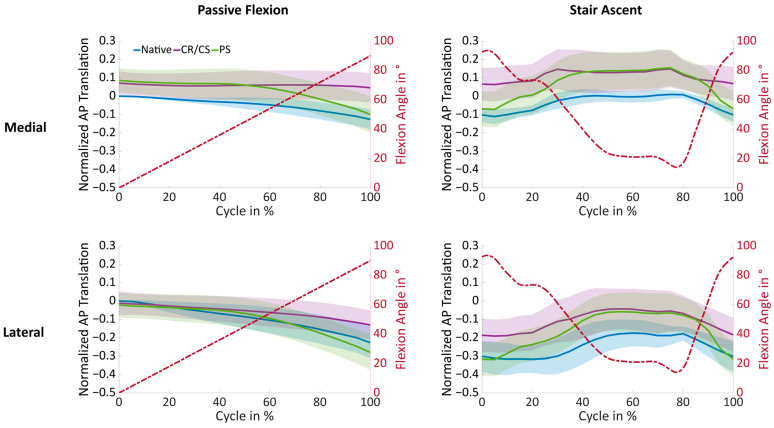
Mean normalized medial and lateral AP translation and standard deviation versus the movement cycle for passive flexion and stair ascent (*n* = 9) in the native condition (blue) and after implantation of the CR/CS (purple) and PS (green) TKA design.

Figure 6 shows the mean normalized medial and lateral AP translation and standard deviation during passive flexion and stair ascent in the native condition and after implantation of the CR/CS and PS TKA designs. Statistical significance is presented in Table 1. For clarity, the condition without cruciate ligaments is not illustrated in this section.

In the native condition, posterior femoral rollback was observed both medially and laterally during passive flexion. After implantation, the medial condyle showed a more anterior position compared to the native knee over the entire range of flexion. The differences in position between the native condition and the CR/CS TKA design remained statistically significant over the entire range of flexion, whereas the differences between the native condition and the PS TKA design were significant only up to 65° of flexion, and then showed almost the same position in the anterior–posterior direction. The lateral condyle generally showed more posterior femoral translation than the medial condyle across all conditions. Furthermore, for the lateral condyle no significant differences were found between the native condition and the PS TKA design over the entire range of flexion. In contrast, the AP position of the CR/CS TKA design differed significantly from the native condition beyond 80° of flexion.

During stair ascent, both condyles showed significant differences in the AP position with the CR/CS TKA design compared to the native condition over the entire range of motion, whereas the PS TKA design differed only at flexion angles below 80°. At flexion angles above 60°, the PS TKA design induced significantly more posterior femoral rollback with both loading profiles. This shows that both loading profiles are capable of revealing differences between the two TKA designs. As expected, no TKA design was superior in recreating the mean native AP translation, which may be due to the high variability in the native knee kinematics and the need for individual selection of the TKA design to restore the individual physiological knee function. Therefore, the kinematic patterns of three exemplary specimens were investigated in more detail in the following.

### 3.3. Individual Kinematic Patterns

**Figure 7:**
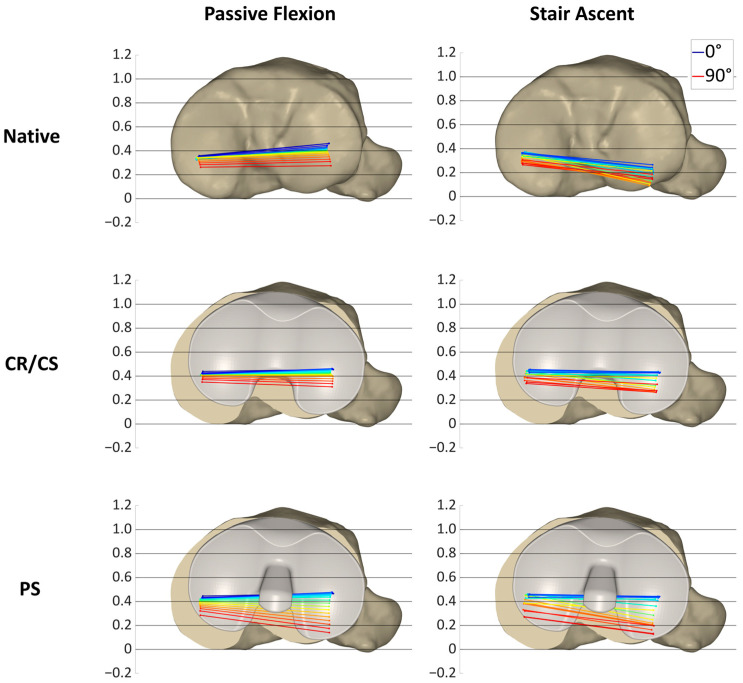
Projection of the flexion axis and the MFC and LFC for specimen S1, showing the individual condylar motion during passive flexion (0°–90° flexion) and stair ascent (16°–93° flexion) for the native condition and after implantation of the CR/CS and PS TKA designs. The colors represent the respective flexion angle in 5° intervals, from dark blue (0°) through green and yellow to red (90°).

**Figure 8:**
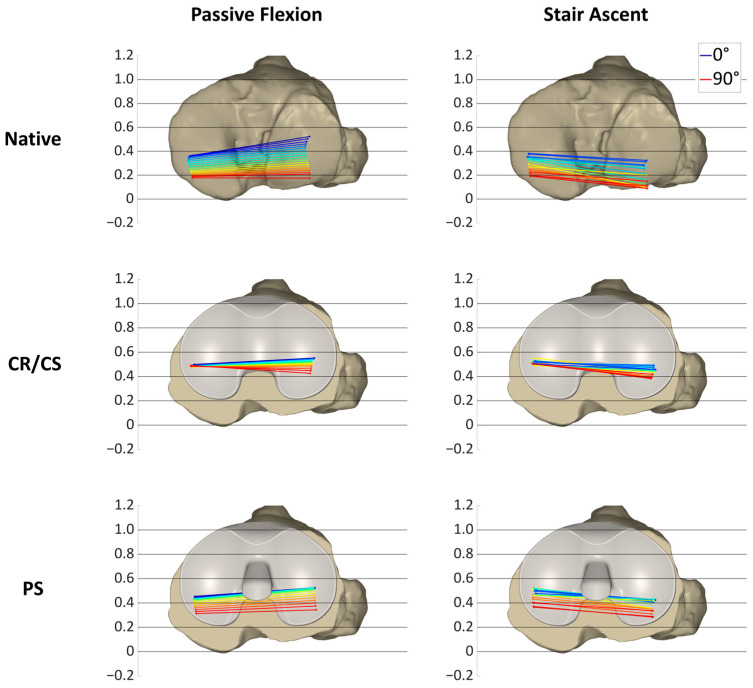
Projection of the flexion axis and the MFC and LFC for specimen S2, showing the individual condylar motion during passive flexion (0°–90° flexion) and stair ascent (16°–93° flexion) for the native condition and after implantation of the CR/CS and PS TKA designs. The colors represent the respective flexion angle in 5° intervals, from dark blue (0°) through green and yellow to red (90°).

**Figure 9:**
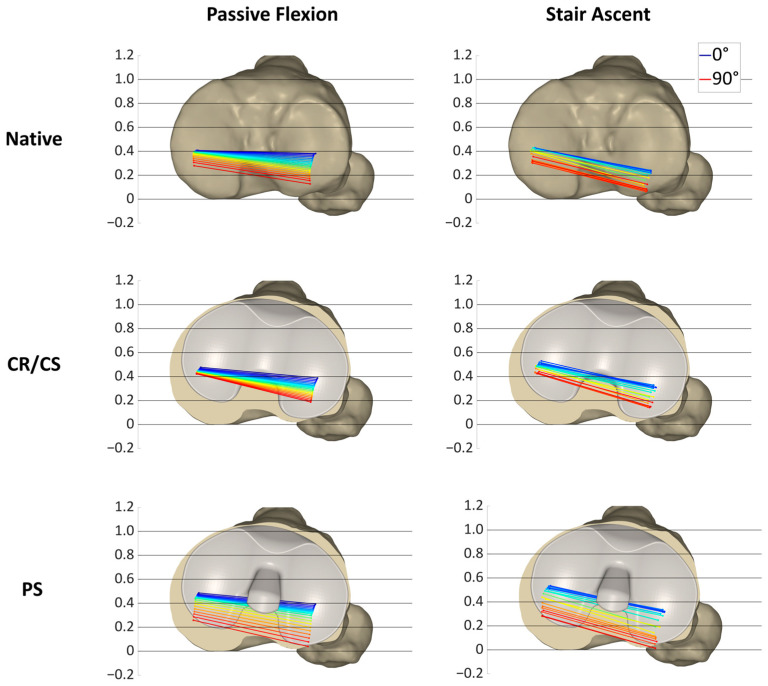
Projection of the flexion axis and the MFC and LFC for specimen S3, showing the individual condylar motion during passive flexion (0°–90° flexion) and stair ascent (16°–93° flexion) for the native condition and after implantation of the CR/CS and PS TKA designs. The colors represent the respective flexion angle in 5° intervals, from dark blue (0°) through green and yellow to red (90°).

Figure 7, Figure 8 and Figure 9 show the projections of the flexion axis and the MFC and LFC of three different specimens throughout the full range of motion for both loading profiles in the native condition and after implantation of the CR/CS and PS TKA designs at 5° flexion and 5% gait cycle intervals, respectively, and thereby reflect the individual condylar motion. The colors represent the respective flexion angle in 5° intervals. The 0° flexion is colored in dark blue and 90° flexion is colored in red. Gait intervals are colored based on the corresponding flexion angle. For consistency, tibia sizes are still standardized. Therefore, 0 and 1 correspond to the most posterior and anterior positions, respectively, regardless of the different tibia sizes.

Figure 7 illustrates the condylar motion of specimen S1 at various conditions and loading profiles. In the native condition, the medial condyle showed approximately the same kinematic pattern for both loading profiles. In contrast, the lateral condyle was positioned approximately 0.2 more posteriorly during stair ascent. With the CR/CS TKA design, the medial condyle shifted approximately 0.1 anteriorly during both loading profiles compared to the native condition. During passive flexion, the lateral condyle showed the same position throughout the range of flexion as in the native condition. During stair ascent, the lateral condyle shifted approximately 0.2 anteriorly for the entire range of motion compared to the native condition, and thus, resulted in the same position as during passive flexion. With the PS TKA design, in extension, the medial and lateral condyles were in the same position as with the CR/CS TKA design for both loading profiles. In flexion, both condyles showed a greater femoral rollback compared to the CR/CS TKA design and the native condition. The most posterior position of the medial and lateral condyles was similar for both loading profiles.

In this specimen, the CR/CS TKA design was superior in recreating the native kinematic pattern, but not the exact kinematics, including the exact AP position and translation. Furthermore, comparable kinematic patterns were observed between the passive flexion and stair ascent after implantation of the TKA components.

In Figure 8, the condylar motion of specimen S2 is presented for various conditions and loading profiles. As for specimen S1, the medial condyle showed approximately the same kinematic pattern for both loading profiles in the native condition, whereas the lateral condyle was positioned more posteriorly during stair ascent. With the CR/CS TKA design, the medial condyle shifted approximately 0.1 anteriorly in extension during both loading profiles compared to the native condition and showed almost no change in position throughout the range of flexion. During passive flexion, the lateral condyle was located approximately in the same position as in the native condition in extension, but exhibited less posterior femoral rollback in flexion. During stair ascent, the lateral condyle shifted approximately 0.15 anteriorly in extension, and it also exhibited considerably less posterior femoral rollback in flexion compared to the native condition. After the implantation of the PS TKA design, the medial and lateral condyles in extension were located almost in the same position as with the CR/CS TKA design for both loading profiles. In flexion, both condyles showed a considerably higher femoral rollback compared to the CR/CS TKA design, but less femoral rollback than in the native condition. The most posterior position of the medial condyle was similar for both loading profiles, but more posterior for the lateral condyle during stair ascent.

For this specimen, the PS TKA design was superior in recreating the posterior femoral rollback as observed in the native kinematic pattern. Furthermore, comparable kinematic patterns were observed between passive flexion and stair ascent after implantation of the TKA components.

Figure 9 shows the condylar motion of specimen S3 at various conditions and loading profiles. In the native condition, the medial condyle showed approximately the same kinematic pattern for both loading profiles, whereas the lateral condyle was positioned more posteriorly and exhibited less femoral rollback during stair ascent. This resulted in a medial pivoting characteristic for passive flexion and a more parallel rollback for stair ascent. With the CR/CS TKA design, the medial condyle shifted approximately 0.1 anteriorly during both loading profiles compared to the native condition. In extension, the lateral condyle showed the same position as in the native condition during passive flexion and was shifted approximately 0.1 anteriorly during stair ascent. Furthermore, the medial and lateral AP ROM during passive flexion was slightly reduced compared to the native condition, whereas the AP ROM during stair ascent remained almost the same. As for the other specimens, with the PS TKA design, in extension, the medial and lateral condyles were in the same position for both loading profiles as with the CR/CS TKA design. In flexion, both condyles showed a considerably higher femoral rollback compared to the CR/CS TKA design and a slightly higher femoral rollback compared to the native condition. As for specimen S1, the most posterior position of the medial and lateral condyles was similar for both loading profiles.

In this specimen, the native kinematic pattern was intermediate between the kinematic patterns of the two different TKA designs. Therefore, both TKA designs are suitable and do not alter the native kinematic pattern substantially.

## 4. Discussion

The main objective of the present study was to compare the AP range of motion and position of the medial and lateral femoral condyles during passive flexion and a complex activity of daily living, in the native condition, after resection of the cruciate ligaments and after implantation of the CR/CS and PS TKA designs. Passive knee flexion and stair ascent did not result in the same AP range of motion for each condition, but showed comparable condylar motion patterns, especially after TKA. As a secondary objective, we investigated whether differences in AP translation between the two different TKA designs can be detected during passive flexion and a complex activity of daily living, and if one design is superior in recreating the AP translation of the native knee. We found significant differences between the TKA designs with both loading profiles, with the PS TKA design showing a higher posterior translation of the femur in flexion. As expected, no design was superior in restoring the mean native AP translation throughout the range of motion. However, when looking at individual specimens, we found that either one of the TKA designs was superior in recreating the individual native kinematic pattern, or that both TKA designs were suitable and did not substantially alter the native kinematic pattern.

To our knowledge, this is the first study that has compared the kinematic patterns of different loading scenarios before and after total knee arthroplasty in the same knees with the same measuring technology in a highly controlled setting. Comparing the same knees at different conditions eliminates the problem of differences between cohorts. In addition, the controlled application of forces along reliable axes is an important aspect to ensure a valid comparison of different conditions in the same knees. Furthermore, using the same definitions while describing kinematics is essential to eliminate potential variations in the results and interpretation of movement [23,24]. This study projected the flexion facet centers (FFCs) of the native femur onto the tibial plane and thereby visualized the kinematic pattern at different conditions. The projection of the FFCs is a widely used method for approximating the tibiofemoral contact situation and is valid for describing the kinematic behavior of the knee [25,26]. However, the projection of the native FFCs after implantation does not reflect the contact situation but the motion that the patient may feel. Another important aspect is the use of the same flexion angle range to compare the AP range of motion and the general kinematic pattern between the loading profiles. This overcomes the influence of the flexion angle on the AP translation, and therefore, only the influence of the loading situation is evaluated [27,28].

The hypothesis that passive and complex loading scenarios show a comparable range of motion in the AP direction, especially after TKA, could not be completely confirmed. In the native condition, the medial condyle showed a comparable AP range of motion and position with both loading profiles. In contrast, the AP range of motion of the lateral condyle revealed significant differences between passive flexion and the stair ascent loading profile in the native condition. Furthermore, the projection of the flexion facet centers showed that the tibiofemoral contact situation also differed between the loading profiles. The lateral condyle was located more posterior during stair ascent than during passive flexion. After cruciate ligament resection, the medial AP range of motion was significantly greater during stair ascent than during passive flexion. In addition, the projections of the MFC and LFC revealed large differences between the general kinematic patterns. During passive flexion, many of the projected points were clustered in one spot, meaning that the condyles were barely moving. This is probably due to the loss of the natural femoral rollback following cruciate ligament resection [29]. Therefore, the AP range of motion was considerably smaller than in the native condition. In contrast, during stair ascent, the projected points showed a large variance. This could be due to the varying degrees of destabilization of the specimens after cruciate ligament resection in combination with the complex loading scenario [20,30,31]. After implantation of the CR/CS TKA design, there were significant differences in the AP range of motion between the loading profiles, both medially and laterally. In contrast, no differences between the loading profiles were found after the implantation of the PS TKA design. This might be due to the higher guidance of the PS TKA design. A previous study of Belvedere et al. also found fewer differences between passive movement and complex activities with a more constraining TKA design [16]. The projection of the MFC and LFC with both TKA designs showed a comparable condylar motion pattern between passive flexion and stair ascent. In addition, a stabilization of the joint with a more anterior position of both condyles during the entire range of flexion was observed during stair ascent compared to the condition after resection of the cruciate ligaments. This demonstrates the generally good performance of the TKA designs and the ability to compare the general motion patterns between passive and complex loading scenarios after TKA. The reason for the large differences in the motion patterns between the loading profiles before TKA could be the high native slope of the specimens and the geometry of the lateral tibial plateau under the influence of the load situation. During stair ascent, the load is considerably higher than during passive flexion, even with load reduced loading profiles. Giffin et al. showed that axial loading in combination with a high tibial slope resulted in a more posterior position of the femoral condyles on the tibia plateau [32]. Furthermore, the complex loading scenarios including AP shear forces combined with the tibial slope may also influence the AP position of the femoral condyles on the tibial plateau. This could explain the more posterior position of the femoral condyles during stair ascent compared to passive flexion before the implantation of the TKA components. However, when looking at the individual specimens, it could be observed that despite the differences between passive and complex loading scenarios, the same TKA design would be chosen to closely mimic the native kinematic pattern.

The hypothesis that with the post-cam TKA design, the femoral posterior translation is higher compared to the TKA design without a post-cam mechanism could be confirmed. At flexion angles above 60°, the PS TKA design induced significantly more posterior femoral rollback for both loading profiles, showing the impact and function of the post-cam mechanism. This demonstrates that both loading profiles were capable of revealing differences between the two TKA designs.

As expected, neither TKA design was superior in restoring the mean native AP translation of both condyles over the entire range of motion. In contrast, when looking at the individual specimens, the CR/CS TKA design was superior in restoring the overall native kinematic pattern in specimen S1, whereas the PS TKA design was superior in specimen S2. This indicates the necessity for individual kinematic analyses and the possibility to select a TKA design out of a comprehensive knee platform to address subject-specific functional needs and thereby restore the physiological knee function. However, neither the exact AP position of the native femoral condyles nor the exact AP translation was restored in these specimens. Given the still relatively high rate of 80% of patients who are satisfied with the results of their TKA, we believe that it is not necessary to exactly restore the native kinematics, but to avoid large differences and restore the general kinematic pattern as observed in the individual specimens. For example, a patient with no femoral rollback in the native condition, but a high femoral rollback after implantation, may not feel comfortable. This is particularly important for characteristic kinematics, such as those in specimens S1 and S2. For specimen S3, both TKA designs may be appropriate as they do not substantially alter the native kinematics. However, as these are only assumptions, further research and clinical studies are needed to identify the relevant parameters that are essential for the restoration of function and patient satisfaction. Furthermore, the extent to which native kinematics should be mimicked needs to be clarified, as they may already be altered due to osteoarthritis. Future studies should also consider the influence of different alignment techniques, soft-tissue balance, tibial slope, and physiological muscle loading to gain a better understanding of the differences in knee kinematics before and after TKA.

The main strength of the present study is the precise measurements of the kinematics under different conditions and loading scenarios in the same knees, which eliminates the problem of large individual and technical differences. However, several limitations should be considered when interpreting the results of this study. First, this study investigated only a small number of human cadaveric specimens that may show variations in soft tissue properties compared to living patients. However, the comparison of passive and complex activities with different implant designs in the same knees is not possible in vivo due to ethical reasons. Second, the applied loading profile used to simulate the complex activity was based on published data on instrumented knee implants [22,33]. This loading profile is also used in complex wear testing and is considered to realistically simulate patient movement under load conditions encountered during daily activities [34]. However, the data were recorded using an ultra-congruent implant design, which may limit their applicability to the intact knee. Therefore, although the applied loading profile resembles an activity of daily living, it does not fully represent the knee movement in all its complexity. However, in the absence of alternatives, the proposed standardized loads appear to be the most appropriate. In addition, the loads were reduced by 75% to avoid specimen damage considering the absence of muscle activity. This is a practice that has been used by others in previous cadaver studies [31,35]. Third, physiological muscle loading was not present and the patellar mechanism was only partially simulated. However, it is assumed that the weight-bearing rather than the muscle contraction has an influence on the tibiofemoral kinematics [18]. Fourth, only flexion angles until 90° were investigated, and kinematic patterns at higher flexion angles may differ. However, the majority of activities of daily living are performed at flexion angles below 90° [36]. Fifth, it was not possible to change the order of the different test conditions. Therefore, time-dependent effects cannot be completely eliminated. Nevertheless, the limited number of tests and the resulting short test duration should minimize these effects [37]. Finally, this study was conducted using a specific TKA design and the results may not be applicable to other TKA designs.

## 5. Conclusions

This study showed that neither TKA design was superior in restoring the mean native AP translation of both condyles throughout the complete range of passive flexion and stair ascent, but that both CR/CS and PS TKA designs may be suitable to restore the individual native kinematic pattern. Moreover, it was shown that passive and complex loading scenarios do not result in exactly the same kinematic pattern, but lead to the same choice of implant design if one design is superior in restoring the general kinematic behavior of the native individual knee. Therefore, this study highlights the importance of individual kinematic analyses to select the appropriate TKA design for a specific patient.

## Figures and Tables

**Table 1:** Statistical significances (*p* ≤ 0.05) of the normalized medial and lateral AP translation between the native condition and the CR/CS TKA design, the native condition and the PS TKA design, and the CR/CS and PS TKA design during passive flexion and stair ascent (*n* = 9). ns = not significant. M = medial. L = lateral.

Passive Flexion							Stair Ascent						
Flexion	Native vs. CR/CS		Native vs. PS		CR/CS vs. PS		% Cycle (Flexion)	Native vs. CR/CS		Native vs. PS		CR/CS vs. PS	
	M	L	M	L	M	L		M	L	M	L	M	L
**0°**	0.024	ns	0.018	ns	ns	ns	**0 (92.6°)**	0.013	0.024	ns	ns	0.009	0.009
**5°**	0.024	ns	0.018	ns	ns	ns	**5 (91.4°)**	0.009	0.018	ns	ns	0.009	0.009
**10°**	0.018	ns	0.024	ns	ns	ns	**10 (81.4°)**	0.009	0.018	ns	ns	0.009	0.009
**15°**	0.018	ns	0.024	ns	ns	ns	**15 (73.7°)**	0.009	0.009	0.044	ns	0.009	0.009
**20°**	0.024	ns	0.024	ns	ns	ns	**20 (73.5°)**	0.013	0.009	0.044	ns	0.009	0.009
**25°**	0.024	ns	0.033	ns	ns	ns	**25 (70.5°)**	0.013	0.009	ns	0.033	0.009	0.009
**30°**	0.024	ns	0.033	ns	ns	ns	**30 (61.1°)**	0.013	0.009	0.024	0.018	0.013	0.009
**35°**	0.024	ns	0.024	ns	ns	ns	**35 (50.5°)**	0.013	0.009	0.024	0.013	ns	0.013
**40°**	0.033	ns	0.024	ns	ns	ns	**40 (40.1°)**	0.013	0.009	0.013	0.009	ns	0.044
**45°**	0.024	ns	0.033	ns	ns	ns	**45 (30.4°)**	0.033	0.013	0.013	0.009	ns	ns
**50°**	0.024	ns	0.033	ns	ns	0.033	**50 (23.7°)**	0.024	0.013	0.013	0.009	ns	ns
**55°**	0.018	ns	0.033	ns	ns	0.024	**55 (21.7°)**	0.013	0.013	0.013	0.013	ns	ns
**60°**	0.013	ns	0.044	ns	0.024	0.013	**60 (21.1°)**	0.013	0.013	0.013	0.018	ns	ns
**65°**	0.013	ns	0.044	ns	0.013	0.009	**65 (21.3°)**	0.013	0.018	0.013	0.018	ns	ns
**70°**	0.009	ns	ns	ns	0.009	0.009	**70 (20.6°)**	0.013	0.013	0.013	0.013	ns	ns
**75°**	0.009	ns	ns	ns	0.009	0.009	**75 (15.6°)**	0.013	0.013	0.013	0.013	ns	ns
**80°**	0.009	0.044	ns	ns	0.009	0.009	**80 (16.9°)**	0.013	0.013	0.013	0.018	ns	ns
**85°**	0.009	0.033	ns	ns	0.009	0.009	**85 (38.7°)**	0.018	0.018	0.018	0.024	ns	ns
**90°**	0.009	0.024	ns	ns	0.009	0.009	**90 (62.5°)**	0.013	0.013	0.033	ns	0.018	0.024
							**95 (83.7°)**	0.013	0.018	ns	ns	0.009	0.009
							**100 (92.5°)**	0.013	0.024	ns	ns	0.009	0.009

## Data Availability

The data presented in this study are available on request from the corresponding author. The data are not publicly available due to ethical and privacy considerations associated with human cadaveric donor material.
